# Product release is rate-limiting for catalytic processing by the Dengue virus protease

**DOI:** 10.1038/srep37539

**Published:** 2016-11-29

**Authors:** A. E. Shannon, M. M. Pedroso, K. J. Chappell, D. Watterson, S. Liebscher, W. M. Kok, D. P. Fairlie, G. Schenk, P. R. Young

**Affiliations:** 1 School of Chemistry and Molecular Biosciences, University of Queensland, St Lucia, Queensland 4072, Australia; 2 ARC Centre of Excellence in Advanced Molecular Imaging, Institute for Molecular Bioscience, University of Queensland, Brisbane, Queensland 4072, Australia

## Abstract

Dengue Virus (DENV) is the most prevalent global arbovirus, yet despite an increasing burden to health care there are currently no therapeutics available to treat infection. A potential target for antiviral drugs is the two-component viral protease NS2B-NS3pro, which is essential for viral replication. Interactions between the two components have been investigated here by probing the effect on the rate of enzyme catalysis of key mutations in a mobile loop within NS2B that is located at the interface of the two components. Steady-state kinetic assays indicated that the mutations greatly affect catalytic turnover. However, single turnover and fluorescence experiments have revealed that the mutations predominantly affect product release rather than substrate binding. Fluorescence analysis also indicated that the addition of substrate triggers a near-irreversible change in the enzyme conformation that activates the catalytic centre. Based on this mechanistic insight, we propose that residues within the mobile loop of NS2B control product release and present a new target for design of potent Dengue NS2B-NS3 protease inhibitors.

Dengue virus (DENV) is an arthropod-borne virus of the family *Flaviviridae*, a large group comprising over 70 human and animal pathogens, including West Nile virus (WNV), Japanese encephalitis virus (JEV) and Yellow fever virus (YFV). According to the World Health Organisation (WHO), approximately 50–100 million cases of infection occur annually, with 2.5 billion people living in high risk regions for epidemic transmission. A recent projection estimated the number of annual infections to be closer to 390 million, over three times greater than previous estimates^1^.

Flaviviruses are small, enveloped viruses with an approximately 11 kb positive-sense RNA genome. The viral RNA is translated from a single open reading frame to produce a single polyprotein precursor. This polyprotein is co- and post-translationally cleaved into three structural (capsid C, membrane prM and envelope E) and seven non-structural (NS1, NS2A, NS2B, NS3, NS4A, NS4B and NS5) proteins. Processing occurs in association with the membrane of the endoplasmic reticulum (ER) and is required for production of functional proteins, and therefore for viral replication. Host cellular proteases, signalase and furin, are responsible for cleaving within the lumen of the ER and within the Golgi, respectively, while cleavage on the cytoplasmic face of the membrane is mediated by a two-component viral protease, comprised of the non-structural proteins NS2B and NS3^2^,^3^,^4^.

The N-terminal 180 amino acids of NS3, termed NS3pro, form a trypsin-like serine protease with a classic His-Asp-Ser catalytic triad^2^,^4^. The catalytic activity of NS3pro is dependent on the presence of a cofactor, NS2B. More specifically, a central hydrophilic domain of NS2B (NS2B_H_), spanning residues 49–95, intimately engages with NS3pro to provide solubility and stability to the protease^5^,^6^,^7^. This hydrophilic domain of NS2B is flanked by three hydrophobic membrane-associating domains which tether NS3pro onto the cytoplasmic face of the ER membrane, where processing occurs. The NS2B-NS3pro complex is required for cleaving internal sites within NS4A and the capsid protein, in addition to sites at the NS2A/NS2B, NS2B/NS3, NS3/NS4A and NS4B/NS5 junctions^4^,^8^,^9^,^10^,^11^,^12^. The absolute requirement of the protease for viral replication and its high conservation throughout the Flavivirus family makes this complex a highly attractive target for inhibitor development. Importantly, the expression of NS3pro with NS2B_H_ alone (lacking the hydrophobic membrane domains of NS2B) is sufficient to yield a soluble and catalytically active protease complex. This recombinant NS2B_H_-NS3pro construct has greatly facilitated *in vitro* drug screening efforts and provides a fast, cost-effective platform for initial drug screens.

The NS2B_H_-NS3pro complex has been crystallised both in ligand-bound and ligand-free forms for several Flaviviruses including DENV^5^,^13^,^14^,^15^,^16^,^17^,^18^. These structures show that NS3pro is comprised of two β-barrels, each formed by six β-strands. One β-strand of the N-terminal β-barrel is provided by the N-terminal region of NS2B_H_ (residues 50–57), which is essential for solubility and stability of the protease complex^5^,^6^. Interestingly, the structure of the C-terminal region of NS2B_H_ (residues 75–95) differs depending on the presence/absence of inhibitors. When ligand-bound, this region forms a β-hairpin loop which is located in close proximity to the active-site of NS3 and assists in the formation of the substrate binding site. In the absence of an inhibitor/substrate, however, this interaction does not occur. The relevance of these crystal structures in terms of drug design is subject to debate, particularly as the hydrophobic membrane domains of NS2B are absent. Nevertheless, the two conformations suggest some degree of flexibility in the C-terminal domain of NS2B_H_.

High-level recombinant expression and purification of NS2B_H_-NS3pro in *Escherichia coli* was first achieved via covalently linking the NS2B_H_ cofactor to the N-terminus of NS3pro with a flexible 9-residue Gly_4_-Ser-Gly_4_ linker domain, hereafter referred to as the ‘glycine linker’^19^. This complex has been used extensively in inhibitor screening assays and crystallisation studies. However, the extent to which the non-native linker-region affects NS2B_H_ flexibility and substrate binding is unknown. Recently, we and others have described methods which allow the co-expression of NS2B_H_ and NS3pro in the absence of a glycine-linker, thus facilitating the production of an unlinked protease complex^20^,^21^. This complex has comparable activity to the enzyme with a glycine-linked equivalent, both in regards to catalytic efficiency and conditions for optimal processing of a short tetrapeptide substrate. However, differences in substrate binding affinity were observed when a hexapeptide substrate was assayed, indicating a potential restriction in accessibility to the substrate binding pocket imposed by the glycine linker^21^.

The majority of current NS2B_H_-NS3pro inhibitor research is focused on targeting the active site of the protease. While standard trypsin-like serine proteases cleave in a position following a single basic residue at the P1 site, the NS2B-NS3pro complex of flaviviruses recognises a dibasic P2-P1 site (Arg or Lys), followed by a short-chain amino acid at the P1′ site (Gly, Ala or Ser)^10^,^11^,^22^,^23^,^24^. This unique specificity could potentially be exploited to allow selective targeting of the viral protease complex. However, despite extensive efforts over the last 15 years, the development of an effective inhibitor has posed significant challenges, many of which can be attributed to the shallow, charged nature of the active site. For this reason, we decided to explore alternative sites of the protease for inhibitor targeting. One such site is at the NS2B cofactor-NS3pro protease interface. In order to gain insight into the role of NS2B_H_ in protease activation, our lab has previously conducted site-directed alanine mutagenesis of the 42-residue NS2B_H_ cofactor domain of the WNV protease^25^. Two sites were identified as being highly important for proteolytic activity, residues 59–62 and 75–85. The latter comprises the flexible, C-terminal region of NS2B_H_. Extending from these earlier studies, we have introduced alanine residues by site-directed mutagenesis to probe specific NS2B_H_-NS3pro interactions important for DENV protease activity. Mutations were introduced in both the glycine-linked and unlinked protease complexes. Three hydrophobic residues distally located from the active site of NS3pro (*i.e.* at the start of the β-hairpin loop) were selected for mutation due to their predicted tight association with NS3pro^5^,^17^,^18^. Two additional residues located within the β-hairpin turn were also chosen to assess differences in flexibility imposed by the artificial glycine-linker. The catalytic properties of these mutant proteases were assessed under both steady-state and single-turnover conditions. This allowed analysis of separate kinetic stages of the enzymatic reaction in order to get a better understanding of the importance of the NS2B_H_ β-hairpin loop in substrate-binding, processing and product release.

## Results

### Expression and purification of mutant constructs

**Figure 1:**
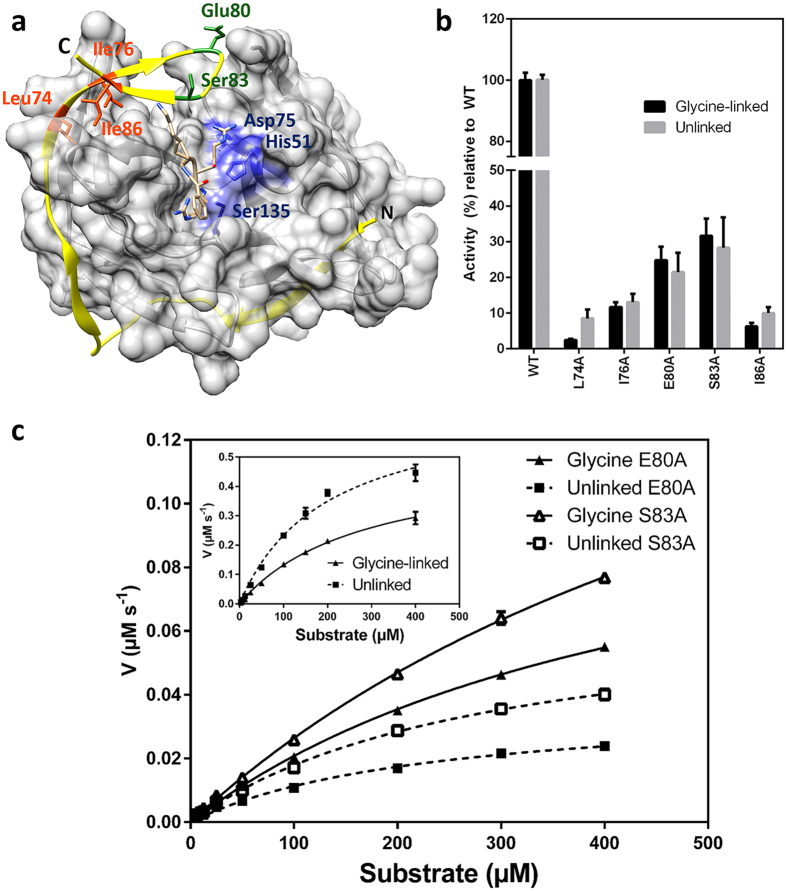
Mutations of NS2B-NS3pro and effect on catalytic activity. (**a**) Crystal structure of DENV3 NS2B_H_-NS3pro in complex with inhibitor Bz-Nle-KRR-H (PDB 3U1I)^17^. The NS3 protease is shown in grey, with the His51-Asp75-Ser135 catalytic triad in blue. NS2B_H_ is shown in yellow with mutated residues shown either in orange (hydrophobic Leu74, Ile76 and Ile86) or green (surface exposed Glu80 and Ser83). Glu80 and Ser83 have been modelled from the native Asp80 and Thr83 present in DENV3 to mimic the DENV2 protein. (**b**) Effect of mutations in NS2B_H_ on the catalytic activity of NS3pro in both glycine-linked and unlinked systems. Activity was measured in 50 mM Tris.HCl, pH 8.5, 37 °C, with 2 μM enzyme (n = 3). Cleavage of the chromogenic substrate Ac-LKRR-pNa (250 μM) was measured by monitoring the change in A_405_ over 10 min. The activity of NS2B_H_ mutants is illustrated relative to that of the respective wild-type forms (**c**) Catalytic activity of mutants and wild-type enzymes measured in 50 mM Tris.HCl, pH 8.5, 37 °C, with 2 μM enzyme (n = 3) as a function of Ac-LKRR-pNa substrate concentration. The progress of the reaction was measured by monitoring the change in A_405_ over 10 min. The data displayed Michaelis-Menten-type behaviour. Main graph: Glu80Ala and Ser83Ala mutants expressed in glycine-linked (solid line) and unlinked (dashed line) systems. Inset graph: Activity of wild-type proteases, reported previously^21^.

The significance of specific residues of NS2B_H_ for the proteolytic activity of NS3pro was evaluated by site-specific mutagenesis. To this end, alanine mutations were introduced into residues Leu74, Ile76, Glu80, Ser83 and Ile86 of the C-terminal β-hairpin of NS2B_H_, which may play an important role in cofactor-NS3 protease interactions (Fig. 1a). Mutation of two of these residues, Leu74 and Ile76, has been reported previously for DENV, and resulted in impaired autoproteolysis and decreased catalytic turnover when assayed under steady-state, Michaelis-Menten conditions^26^. We have included these residues in our study to probe the specific stage of the enzymatic reaction they may be involved in. Mutations were introduced into both the glycine-linked NS2B_H_-NS3pro and the unlinked equivalent in order to assess the effects of the non-native linker on catalytic processing. Recombinant proteins were expressed in *E. coli* and purified from the soluble fraction with Ni^2+^-affinity chromatography, utilising the His_6_ tag on NS2B_H_. High yields of each mutant were obtained from the soluble fraction for both constructs, suggesting that the introduced mutations do not affect native folding of the proteins (see supplementary Fig. S1). Yields of soluble protein obtained following purification were comparable between the glycine-linked and unlinked systems, and ranged from 10–30 mg/L of *E. coli* culture.

### Steady-state catalytic properties of NS2B-NS3pro mutants

The proteolytic activity of the mutants was determined by monitoring cleavage of the chromogenic *para*-nitroanilide substrate Ac-LKRR-*p*Na. This was initially performed using a single substrate concentration (250 μM), with cleavage monitored over 10 min. Each of the NS2B_H_ mutations decreased activity to ≤30% relative to the wild-type protease in both the unlinked and glycine-linked complexes (Fig. 1b). The most significant decrease was observed for mutations of the hydrophobic residues Leu74, Ile76 and Ile86. The three residues are located in a hydrophobic cleft formed by NS3pro, which permits engagement of the β-hairpin loop of NS2B_H_ (consisting of residues 74–86) with the active site of the protease (Fig. 1a). Leu74 and Ile76 are situated at the start of the loop, while Ile86 is located at the end. Therefore, it is apparent that these hydrophobic residues are essential for catalytically relevant interactions between the β-hairpin loop and the active site of the protease.

In contrast, according to the crystal structure^17^ the side-chains of the hydrophilic residues Glu80 and Ser83 are facing away from the active site (Fig. 1a). The relevant catalytic parameters (*i.e.* k_cat_, K_m_ and k_cat_/K_m_) for these mutants were determined by measuring catalytic rates at a range of substrate concentrations (Fig. 1c) and the data were fitted to the Michaelis-Menten equation (eq. 1). Relevant parameters are summarised in Table 1. For the mutants of the three hydrophobic residues, the low turnover number and high K_m_ value prevented a similar analysis.

Mutation of residues Glu80 and Ser83 lead to a significant decrease of k_cat_ when compared to the values measured for the corresponding wild-type systems (Table 1). However, the effect of the mutations is more pronounced in the unlinked system, where the catalytic rate is reduced to less than 10% of that of the corresponding wild-type. In contrast, the effect of the mutations on substrate binding is less significant (estimated from a comparison of the respective K_m_ values), in particular for the unlinked system. When comparing overall catalytic efficiency (k_cat_/K_m_), mutations introduced into the unlinked system had a considerably greater effect, with only ~5% residual efficiency, compared to ~15% in glycine-linked mutants. It is possible that the glycine-linker is providing a structural rigidity that renders this system more resistant to the effect of the mutations. As the non-native linker may promote the interaction between NS2B_H_ and NS3pro, cleavage of the substrate may occur more efficiently for this construct despite a decreased substrate affinity. Nevertheless, both systems show consistency in the overall effect of mutating different residues, the greatest decrease in activity being observed for the three hydrophobic residues.

**Figure 2:**
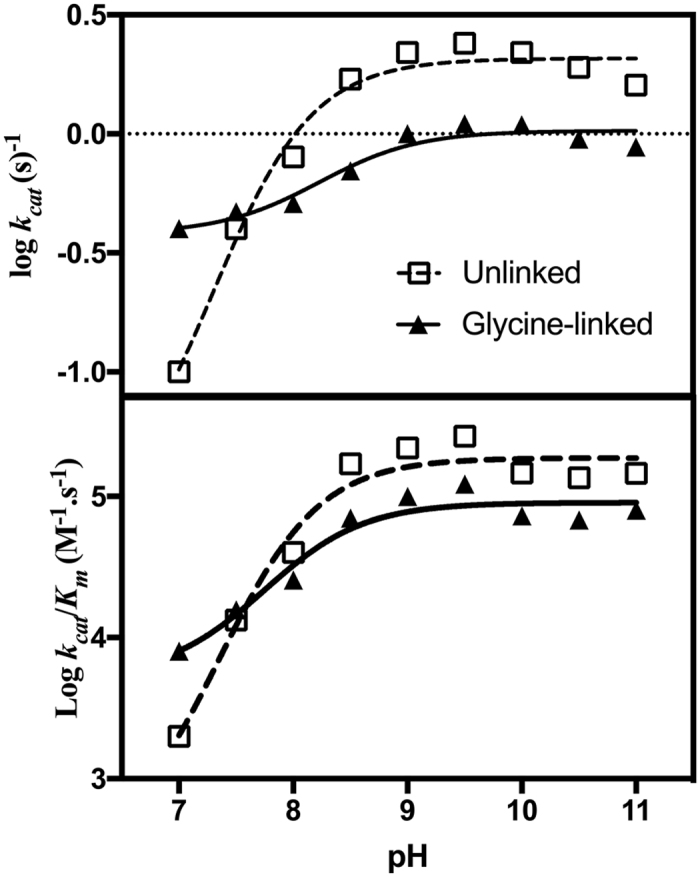
The effect of pH on the catalytic parameters of wild-type linked and unlinked NS2B_H_-NS3pro complexes. Catalytic rates (top panel) and catalytic efficiencies (bottom panel) were recorded at pH values ranging from 7.0 to 11.0 for both the unlinked (□) and glycine-linked (▲) proteasess. Activity was measured by monitoring hydrolysis of Ac-LKRR-*p*Na substrate at 37 °C in a multicomponent buffer (100 mM acetate, 100 mM MES, 100 mM HEPES, 100 mM CHES, 100 mM CAPS). The pH profiles were fitted using an equation (eq. 2), derived for a monoprotic system where the fully protonated species is either catalytically inactive (i.e. κ = 0) or partially active (κ = 0.31 ± 0.03)^32^,^43^.

To determine whether the glycine-linker affects the catalytic mechanism of wild-type NS2B_H_-NS3pro complexes, an investigation of the effect of pH on k_cat_ and k_cat_/K_m_ of the two systems was carried out (Fig. 2, Table 2). Flavivirus proteases, including DENV, characteristically reach a maximum activity at pH > 9.0^19^,^21^,^27^. The observed pH dependence (Fig. 2) is characteristic for systems where a minimum of one protonation equilibrium contributes to both the rate (i.e. pK_es1_) and the catalytic efficiency (i.e. pK_e1_)^28^,^29^,^30^,^31^,^32^. The main difference between the unlinked and glycine-linked systems is that for the former the activity at low pH continues to decrease while for the latter it reaches a final value (at pH 7 the respective rates are 0.1 s^−1^ and 0.4 s^−1^; Fig. 2). Consequently, the two data sets were analysed using equation 2, with κ values of either 0, or an estimated value of 0, respectively (see Materials and Methods section for the definition of κ).

The assignment of the pK_a_ values to specific residues within the active site of the NS2B_H_-NS3pro complex is not trivial due to the complexity of the active site. However, some predictions can be made based on the well-described catalytic mechanisms of other serine proteases, particularly chymotrypsin, which also utilises a classic Ser-His-Asp catalytic triad^33^,^34^. Adopting the model proposed for the reaction mechanism employed by chymotrypsin, the first stage of the NS3pro-catalysed reaction involves a nucleophilic attack on the carbonyl carbon of the peptide by the Ser residue of the triad (*i.e.* Ser135 in Fig. 1a). The nucleophilicity of this residue is greatly enhanced by its deprotonation, whereby the triad His (*i.e.* His51; Fig. 1a) acts as a general base. The aspartate in the catalytic triad (*i.e.* Asp75; Fig. 1a) forms a hydrogen bond with this histidine, an interaction that may raise the pK_a_ of this residue^33^,^35^. We therefore ascribe pK_e1_ and pK_es1_ to this histidine (Table 2).

### Catalytic activity of NS2B-NS3pro mutants under single-turnover conditions

**Figure 3:**
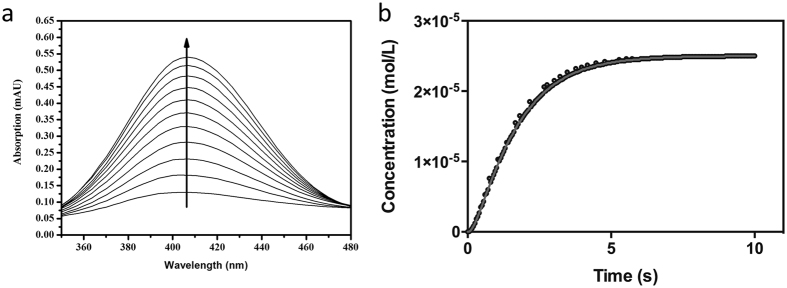
Single turnover enzyme kinetics. Hydrolysis of the *para*-nitroanalide substrate Ac-LKRR-*p*Na (25 μM) by the wild-type form of the glycine-linked DENV2 protease complex NS2BglyNS3pro. Activity measured in 50 mM Tris.HCl, pH 8.5 at 37 °C. (**a**) Product formation monitored by diode array between 310-725 nm. For illustration only data measured in 50 ms intervals are shown. The arrow indicates increasing product concentrations at optimal λ = 405 nm (Ɛ_405_ = 9,500 M^−1^cm^−1^). (**b**) Time course for product formation. The data were fitted to a first-order exponential (providing an estimate of k_obs_) using Reactlab software (eq. 3). Corresponding data for the unlinked wild-type system and the various mutants tested are shown in the Supplementary Section (see supplementary Fig. S3).

In the previous section we demonstrated that mutations within the flexible C-terminal region of NS2B_H_ are detrimental to both catalytic rate (*i.e.* k_cat_) and substrate interaction (*i.e.* K_m_). In order to better understand the effect of these mutations on catalysis, and to determine the rate-limiting step of the reaction, single turnover experiments were carried out. Progress of substrate cleavage was monitored at 405 nm with a diode array over a period of 10 s (Fig. 3).

Data were recorded for the five mutants in the flexible C-terminal region of NS2B_H_, for both the glycine-linked and unlinked complexes, and fit to a first order exponential (eq. 3). All data were obtained using a 20% excess of enzyme, however in order to confirm that this excess was sufficient to satisfy single-turnover requirements, additional data were also obtained for the wild-type form of the glycine-linked enzyme with increased enzyme concentrations. The ratio of enzyme to substrate was varied from 1.2 to 2.8 and 4.6 (enzyme excess from 20% to over 360%), using a maximum enzyme concentration of 70 μM. The resulting rate constants varied by less than 5%, thus demonstrating that a 20% excess of enzyme adequately represents single turnover conditions.

Resulting rate constants (k_obs_) are summarised in Table 3 and indicate relatively small variations between the different systems. Since the rate constants describe the process starting with binding of the substrate and ending with the conversion of the enzyme-substrate (ES) complex to the enzyme-product (EP) complex it is evident that the wild-type forms of the glycine-linked and unlinked systems operate virtually identically with k_obs_ ~0.85 s^−1^. This observation is consistent with the hypothesis that both systems utilise a conserved mechanistic strategy. Since k_obs_ is also approximately two- to three-fold higher than k_cat_ (Table 1), it emerges that the substrate cleavage (with the concomitant release of the chromogenic *p*Na) is not rate-limiting.

A similar observation has been reported for serine proteases, including chymotrypsin when assayed using a *p*-nitrophenyl acetate as substrate. Upon substrate addition the enzyme displays a pre-steady state ‘burst’ phase associated with rapid formation of the ES complex and subsequent release of *p*-nitrophenyl, followed by the rate-limiting hydrolysis and release of the covalently bound reaction intermediate^36^. More recently a similar biphasic reaction was reported for the Prostate-Specific Antigen (PSA) serine protease^37^. It is therefore likely that the DENV2 protease-catalysed reaction proceeds in a similar manner, whereby the rate-limiting step may be related to the product release and/or the regeneration of the active site. Furthermore, this difference is more significant in the mutant complexes than for the respective wild-type forms, with k_obs_ ~10–30 times greater than k_cat_ for mutant complexes (compare Tables 1 and 3). Hence, while the mutations cause a considerable loss in catalytic activity, this loss is not associated with substrate binding or the substrate cleavage of the reaction. It thus emerges that residues in the flexible loop may play an important role in the final stages of the catalytic cycle (*i.e.* product release and/or the regeneration). This is particularly the case for the three hydrophobic residues flanking the beginning and end of the loop (*i.e.* Leu74, Ile76 and Ile86).

### Analysis of conformational changes by tryptophan fluorescence

**Figure 4:**
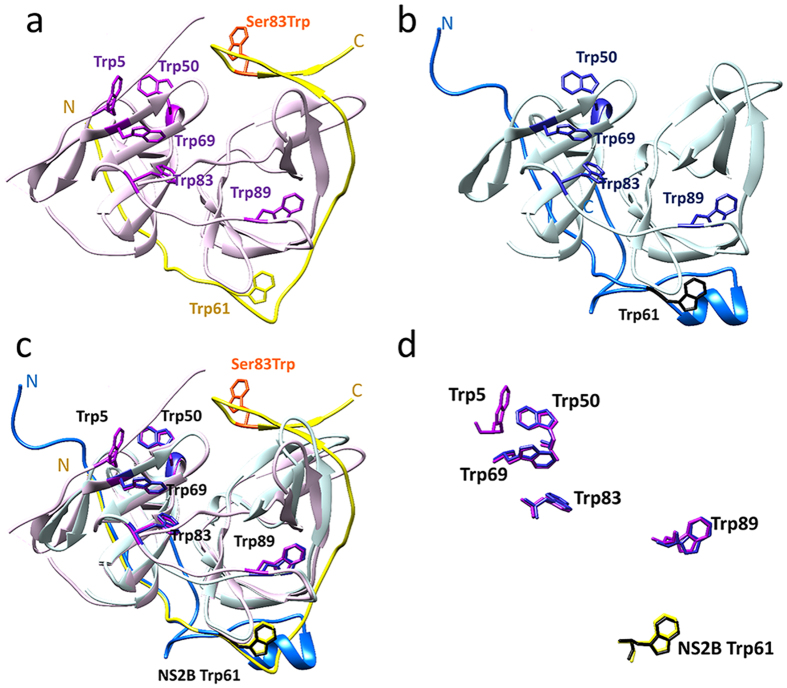
Crystal structure of the DENV NS2B_H_-NS3 protease showing the location of tryptophan residues. (**a**) DENV-3 protease in complex with Bz-Nle-KRR-H inhibitor (PDB 3U1I)^17^. NS2B_H_ is shown in yellow and NS3pro in purple. The Ser83Trp mutation is shown in orange. (**b)** Ligand-free DENV-2 (PDB 2FOM-1)^5^. NS2B_H_ is shown in dark blue and NS3pro in light blue. Ser83 of NS2B_H_ and Trp5 of NS3pro are unresolved in the crystal structure. **c)** Alignment of ligand-bound and ligand-free NS2B_H_-NS3pro. In the ligand-bound structure, the C-terminal region of NS2B_H_ (yellow) forms a β-hairpin which wraps around NS3pro. In the ligand-free structure, the C-terminal domain of NS2B_H_ (dark blue) does not engage with NS3pro. (**d**) Alignment of wild-type tryptophan residues of NS2B_H_ in yellow and black, and NS3pro in purple and blue for ligand-bound and ligand-free complexes respectively. Trp5 was unresolved in the ligand free crystal structure.

The kinetic data discussed in the previous sections indicate that (i) the glycine linker only has a modest effect on the catalytic properties of the protease and (ii) the major role of the flexible hairpin loop is associated with product release and/or active site regeneration. However, although the data above support a mechanistic model whereby this loop does not play an important role in substrate binding, available crystal structures of the NS2B_H_-NS3pro complexes from WNV and DENV have shown that the loop may adopt distinct conformations dependant on whether the complex is ligand-bound (with the ligand benzoyl-norleucine-Lys-Arg-Arg-H) or ligand-free (Fig. 4a and b)^5^,^17^. We thus used tryptophan fluorescence spectroscopy to evaluate if movements of the β-hairpin loop are associated with the catalytic cycle of the NS2B_H_-NS3pro complex. In NS2B_H_ only one tryptophan residue (Trp61) is present, located within the N-terminal β-sheet and adjacent to the hairpin loop. In both the ligand-bound and ligand-free crystal structures of WNV and DENV, the side chain of Trp61 adopts a similar orientation, suggesting minimal movement. There are also several tryptophan residues in NS3pro that also maintain conserved conformations independent of the absence or presence of ligands (Fig. 4c and d). Therefore, to specifically look at the movement of the β-hairpin loop, site-directed mutagenesis was used to introduce a tryptophan residue into this region. Since the replacement of Ser83 by alanine had the least significant effect on catalysis (Table 1) this site was chosen to introduce a tryptophan (Fig. 4a). Interestingly, while the unlinked Ser83Trp mutant behaved virtually identically to the wild-type enzyme, the glycine-linked mutant displayed a greater catalytic turnover rate (k_cat_), at the likely cost of reduced substrate affinity (estimated from a comparison of the respective K_m_ values; Table 4). Nonetheless, the overall catalytic efficiencies (k_cat_/K_m_ M^−1^s^−1^) for mutants of both linked and unlinked complexes were similar to their respective wild-type forms (Table 4), and therefore were used for the analysis of the movement of the β-hairpin loop.

**Figure 5:**
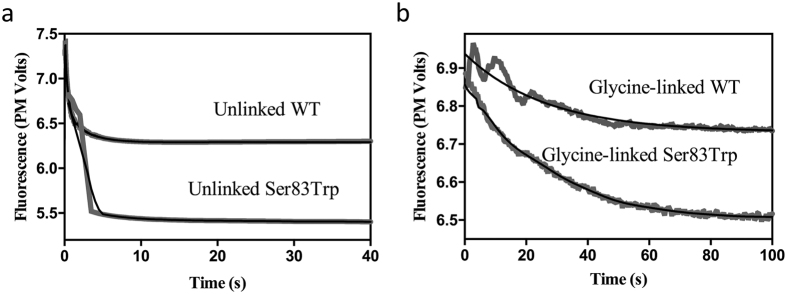
Time course of fluorescence changes. Changes in fluorescence recorded upon the addition of Ac-LKRR-*p*Na substrate (25 μM) for both the glycine-linked and unlinked systems, for their wild-type (WT) and Ser83Trp mutant forms. Fits to the data are shown in black (**a**) Unlinked enzymes (**b**) Glycine-linked enzymes.

The time course of fluorescence changes upon the addition of substrate was recorded for both the glycine-linked and unlinked systems, for their wild-type and Ser83Trp mutant forms (Fig. 5). For the unlinked systems a rapid quench is observed, complete in less than 5 s. For the glycine-linked systems the rate of quenching is considerably slower. The data were analysed by fitting to exponential equations (eqs 3 and 4).

Analysis of the fluorescence data suggests that both the glycine-linked and unlinked wild-type enzymes can be characterised by a single exponential decay with the rate constant k_1_, which describes structural changes upon adding substrate. In contrast, for the Ser83Trp mutants of these systems the corresponding fluorescence changes are best described by a double-exponential decay (characterised by rate constants k_1_ and k_2_). The structural changes associated with k_1_ are of comparable magnitude in both linked and unlinked systems, independent of the Ser83Trp mutation, since the change in rate constant is ~12–15 s^−1^ for the linked and ~50–100 s^−1^ for the unlinked system (Table 4). It is therefore plausible to associate this transient to a perturbation caused by interactions between the substrate and any of the native tryptophan residues present in the NS2B_H_-NS3pro complex. As Trp5 of NS3pro (Fig. 4) is not resolved in the ligand-free crystal structure, it is possible that this residue may be associated with this transient. In contrast, k_2_, only observed in the Ser83Trp mutants of both systems, may thus be associated with perturbations affecting Trp83. For both the linked and unlinked systems k_2_ is considerably larger than k_1_. Thus, the fluorescence data indicate that the initial binding of substrate to the active site (associated with k_1_) is followed by a rapid movement of the β-hairpin loop (associated with k_2_), possibly leading to a closure of the catalytic centre (compare Fig. 4a and b). This is in agreement with the observation that mutations of residues E80 and S83 lead to an increase in K_m_ values (Tables 1 and 4).

Importantly, for all systems investigated, the quenched fluorescence does not recover (Fig. 5), even after 1000 s (data not shown), indicating that structural changes induced by substrate binding appear to be irreversible on the time scale employed for the catalytic measurements. Previous data have shown that cleavage products can remain bound to the active site in NS3pro^17^,^38^, possibly supported by stabilising electrostatic interactions. It therefore seems likely that the hydrolysis products may remain bound to the protease until displaced by the next incoming substrate molecule. As a result, the enzyme complex does not return to its starting open conformation after completion of a single catalytic cycle. This interpretation is consistent with product release being the rate-limiting step for the catalytic turnover, and can be compared to the catalytic mechanism of the serine protease chymotrypsin.

Substrate hydrolysis by chymotrypsin is commonly referred to as a two-stage ‘ping-pong’ mechanism. The first stage involves an attack on the carbonyl carbon of the substrate by the catalytic serine, forming a short-lived tetrahedral intermediate. This quickly collapses resulting in cleavage and release of the C-terminal part of the substrate. As a result, an acyl-enzyme intermediate is temporarily formed, with an ester bond between the oxygen of the active-site serine and the carbonyl carbon of the N-terminal fragment of the substrate. In the second step, the remaining ester is hydrolysed by a water molecule that is activated by the His51-Asp75 catalytic diad. During this two-stage reaction, the protein conformation is thought to change. It is likely that the DENV protease operates in a similar manner (see supplementary Fig. S2), with a conformational change in the enzyme required for processing. The cofactor is likely to be influential in this transition, particularly in enabling release of the products.

## Discussion

We have conducted a comprehensive analysis of the catalytic behaviour of a recombinant dengue virus protease under single turnover and steady-state conditions. This analysis has shown that the release of the product from the active site is rate-limiting, and that residues within the mobile β-hairpin loop of NS2B_H_ play an essential role in product release. Although this loop forms one face of the substrate-binding cleft, mutations within this region do not greatly affect the initial phase of the catalytic cycle. Of particular relevance are the three hydrophobic residues that define the beginning and end of the β-hairpin loop (*i.e.* L74, I76 and I86). Their replacement by alanine leads to catalysts with minimal residual activity when assayed under steady-state conditions (Fig. 1b). However, under single-turnover conditions their catalytic potential appears only mildly impaired (Table 3). Therefore, it appears these residues contribute to product release and regeneration of the active site for subsequent rounds of hydrolysis. Both linked and unlinked NS2B_H_-NS3pro complexes were compared and, while mutation of the β-hairpin loop in both systems showed essentially the same effect, this was more pronounced in the unlinked NS2B_H_-NS3pro. This is consistent with previous data, which suggest that while the two systems operate similarly, the artificial linking of NS2B_H_ and NS3pro may have subtle effects on proteolytic activity^21^. This interpretation is further supported by the minor differences we observed when probing the effect of pH on the catalytic performance of the two systems (Table 2) and suggests the unlinked system to be a better model for enzyme activity.

Monitoring the change of tryptophan fluorescence under single turnover conditions indicated that the protease undergoes a fast conformational change upon the addition of substrate, but does not return to its initial state (Fig. 5). This observation further substantiates the hypothesis that product release is the rate-limiting step in the catalytic cycle. This interpretation is further supported by structural data^17^,^38^. In the first reported ligand-bound DENV crystal structure, two different complexes were observed in the asymmetric unit, one with the full tetrapeptide inhibitor (bz-Nle-KRR-H) and one with a degraded peptide, which was modelled as a di-Arg degraded product^17^. Despite minimal contact with NS2B_H_ and NS3pro, the di-Arg was sufficiently stabilised to remain bound close to the active site.

Crystal structures of both WNV and DENV show that the protease may adopt two distinct conformations^5^,^14^,^17^,^18^. While the ligand-bound ‘closed’ active conformation is well accepted, the structure of the ligand-free form has been the subject of debate. Available crystallographic data suggest that the β-hairpin of NS2B_H_ may be relatively flexible and directed towards the solvent, forming no interactions with NS3pro^5^. The observation of extreme broadening of NMR resonances supports structural instability of the ligand-free complex and the potential for the β-hairpin to adopt varied conformations^20^,^38^,^39^,^40^. Alternatively, NMR studies utilising ^15^N-relaxation rates, paramagnetic relaxation enhancement (PREs) and pseudo-contact shifts (PCS) in the unlinked protease complex have indicated that the crystallised ‘open’ conformation of DENV protease is instead a result of destabilisation or degradation of the complex caused by high pH and ionic strength^20^,^38^. These studies suggest a higher prevalence of the closed-conformation in solution, even in the absence of an inhibitor. However, the observed change in tryptophan fluorescence associated with residue Trp83 in the β-hairpin loop indicates that substrate binding triggers significant conformational changes, consistent with the hypothesis that in the absence of ligand this region is not ‘closed’. While the precise conformation of the ligand-free β-hairpin loop may differ from the arrangement represented in available crystal structures^5^,^13^, this region exhibits an intrinsic level of flexibility that is associated with the catalytic reaction.

We propose that a more constrained ‘closed’ enzyme conformation is induced upon binding of the first substrate molecule and that this enzyme conformation is closer to the active catalytic form that binds the next substrate molecule after product displacement. The rate-limiting release of the product may be mediated via residues within the β-hairpin loop, especially L74, I76 and I86, possibly by their interactions with an incoming substrate molecule. The substrate-induced conformational change of the NS2B_H_-NS3pro complex, and the role that residues in the β-hairpin loop may play in product release, could be potentially exploited in future inhibitor design for the flavivirus serine proteases. While the majority of current research is focused on developing active site inhibitors, this has met with limited success. Compounds which prevent the interactions between NS2B_H_ and NS3pro have also been suggested^22^,^41^, however, this has been based on the assumption that prevention of correct NS2B_H_ β-hairpin engagement would inhibit substrate binding or catalysis. As such, computational screening of compounds directed at inhibiting NS2B_H_ binding would have been undertaken with models based on the ligand-free NS3 active site. However, the results presented here have shown that substrate binding triggers considerable structural rearrangement of the NS2B_H_ β-hairpin loop. Computational docking studies might therefore be more productive if targeted at ligand-bound conformations of the enzyme. Promising inhibitors could act by stabilising the product-bound enzyme conformation or by blocking product release rather than substrate binding, and thereby inhibiting the catalytic cycle. Interestingly, this approach has been used for aspartic proteases where a higher affinity constrained bicyclic substrate was found crystallographically to be locked in the enzyme active site in its cleaved form, the products dissociating much more slowly than those derived from native substrate components^42^. Although some potent inhibitors of flavivirus proteases that target the substrate-binding catalytic site have been obtained to date, they have so far failed to progress to the clinic and new approaches might prove to be more successful. Here, we have described a novel finding that Dengue NS2B-NS3 protease-catalysed substrate processing is affected by product release and that the product-bound enzyme conformation may be different to the substrate-binding conformation and this may be an important clue for alternative inhibitor design. The stabilization of this product-bound conformation of the enzyme may be an alternative and innovative strategy for inhibitor development that warrants further investigation. Efforts towards realizing this strategy are currently in progress.

## Methods

### Mutant constructs

The expression plasmids pQE9 NS2B_H_^49–95^-Gly_4_SerGly_4_-NS3pro^1–185^ (NS2B_H_-gly-NS3pro, glycine-linked) and pETDUET-1 NS2B_H_^49–95^ + NS3pro^1–185^ (unlinked), as previously described^21^, were used as the templates for mutagenesis of the glycine-linked and unlinked protease complexes respectively. Site-directed alanine mutations were introduced using partially overlapping, outward-facing primers for whole-plasmid PCR amplification with Phusion polymerase (NEB). Following PCR, template plasmid was digested with DpnI (NEB) for 1 hr at 37 °C. DH5α *E. coli* were transformed with digested products and grown on LB agar plates in the presence of ampicillin (100 μg/ml) for pETDuet and PQE9. Correct mutagenesis was confirmed through Sanger sequencing at the Australian Genome Research Facility (AGRF) Brisbane.

### Expression and Purification

Recombinant pETDuet NS2B_H_-NS3pro and pQE9 NS2B_H_-gly-NS3pro mutant plasmids were transformed into BL21(DE3) and SG-1009(pRep4) *E. coli* respectively for expression, as previously described^21^. Briefly, 500 mL cultures of each mutant were grown until the OD_600_ reached 0.5. Protein was expressed for 3 hrs at room temperature following induction with 0.4 mM isopropyl β-D-thiogalactopyranoside (IPTG). Following expression, cells were pelleted, resuspended in ice cold lysis buffer (50 mM NaH_2_PO_4_ pH 7.4, 300 mM NaCl), lysed via sonication and centrifuged to remove insoluble products. Protein was purified from the soluble lysate through Ni^2+^ affinity chromatography with Ni-NTA resin (GE healthcare) pre-equilibrated with lysis buffer. Protein was bound for 30 min at 4 °C on a rotor and resin washed with wash buffer (50 mM NaH_2_PO_4_ pH 7.4, 300 mM NaCl, 20 mM imidazole) before eluting with 5 ml elution buffer (50 mM NaH_2_PO_4_ pH 8, 300 mM NaCl, 250 mM imidazole). Eluted protein was analysed on a 14% SDS-PAGE gel stained with Coomassie Blue. Protein concentration was determined using a BCA assay as per the manufactures instructions (Thermo Scientific).

### Steady-state kinetic activity analysis

Activity of DENV2 NS2B_H_-NS3pro mutants were compared to the wild-type protease complex by monitoring cleavage of the chromogenic peptide substrate Ac-LKRR-*p*NA, through a previously described spectrophotometric protease kinetics assay^19^. Assays were performed in a 96-well-plate in triplicate, in 50 mM Tris, pH 8.5, with a final enzyme concentration of 1 μM per well. Mutants were initially assayed through measuring change in absorbance at 405 nm over 10 min, with a single substrate concentration of 250 μM. To obtain steady-state kinetic parameters, substrate concentrations were then varied from 6 μM–400 μM with a constant enzyme concentration of 1 μM. Data were fit to the Michaelis-Menten equation (eq. 1), using GraphPad Prism software.


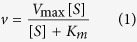


In order to assess the effect of pH on catalytic parameters, assays were carried out under the same steady-state conditions, but in a multicomponent buffer (100 mM acetate, 100 mM MES, 100 mM HEPES, 100 mM CHES, 100 mM CAPS) with pH values ranging from 7–11 in intervals of 0.5 units. The pH profiles were fitted using an equation (eq. 2), derived for a monoprotic system where the fully protonated species is either catalytically inactive (i.e. κ = 0) or partially active (i.e κ = 0.31 ± 0.03)^32^,^43^. The parameter κ describes the ratio of the activities of the partially and fully active forms of the enzyme, H is the proton concentration, *K* represents the acid dissociation constant for either the enzyme-substrate complex (ES) or the free enzyme (E), *c* is the pH independent value of *y*, *y* is the kinetic parameter of interest, *i.e. k*_*cat*_ or *k*_*cat*_/*K*_*m*_.


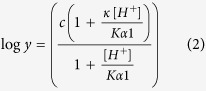


### Single-turnover stopped-flow kinetics

Stopped-flow absorbance and fluorescence experiments were performed using an Applied Photophysics SX-18 spectrometer. All measurements were carried out in quintuplicate in 50 mM Tris.HCl at pH 8.5 and 37 °C, with final concentrations of protein and substrate of 30 μM and 25 μM, respectively (*i.e.* a 20% excess of enzyme). Note that although an ~20% excess of enzyme is commonly used in similar single turnover experiments^30^,^44^ the suitability of the conditions was tested with the wild-type form of the glycine-linked protease by varying the ratio of [E]:[S] from 30:25, 70:25 and 70:15. The resulting rate constants varied by less than 5%, thus demonstrating that a 20% excess of enzyme adequately represents single turnover conditions. The reaction was initiated by mixing equal volumes of enzyme and substrate to a total volume of 65 μL. The path length of the observation cell was 2.0 mm. Fluorescence measurements were carried out with an excitation wavelength of 260 nm and an emission wavelength of 310 nm, and both excitation and emission slits were maintained at 2 mm. The photomultiplier input was adjusted to maintain the total signal at 8 V between the protein in the absence of substrate and the dark current readings. The data were recorded as photomultiplier output in volts. The absorption experiments were recorded with a photo-diode array detector over the wavelength range of 310–725 nm. Rate constants (*k*_*obs*_) were obtained by fitting experimental data to first (eq. 3) or second order exponential (eq. 4) equations, where the parameters A and B are the amplitude of the curve, k is the rate, and c is an independent constant.


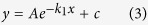



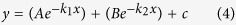


## Additional Information

**How to cite this article**: Shannon, A. E. *et al.* Product release is rate-limiting for catalytic processing by the Dengue virus protease. *Sci. Rep.* **6**, 37539; doi: 10.1038/srep37539 (2016).

**Publisher’s note:** Springer Nature remains neutral with regard to jurisdictional claims in published maps and institutional affiliations.

## Supplementary Material

Supplementary Information

## Figures and Tables

**Table 1:** Kinetic parameters for the hydrolysis of Ac-LKRR-*p*NA substrate by wild-type, E80A and S83A mutants of both the unlinked and Glycine-linked systems.

	K_m_ (*μM)*	k_cat_ (*s*^*−1*^)	k_cat_/K_m_ (_M_^−1^ s^−1^)
Glycine-linked WT	282 ± 22	0.34 ± 0.01	1191 ± 104
Glycine E80A	480 ± 29	0.08 ± 0.01	167 ± 12
Glycine S83A	708 ± 51	0.14 ± 0.01	200 ± 18
Unlinked WT	197 ± 21	0.46 ± 0.02	2345 ± 276
Unlinked E80A	234 ± 19	0.025 ± 0.001	107 ± 10
Unlinked S83A	285 ± 20	0.045 ± 0.002	161 ± 13

Activity measured at 37 °C, pH 8.5 and fitted according to the Michaelis-Menten equation. Wild type data were previously reported^21^.

**Table 2:** Relevant pK_a_ values estimated from the pH dependence of k_cat_ and k_cat_/K_m_.

	pK_es1_	pK_e1_
Glycine-linked	8.6 ± 0.4	8.1 ± 0.3
Unlinked	8.2 ± 0.1	8.7 ± 0.7

Activity measured by monitoring hydrolysis of Ac-LKRR-*p*Na substrate at 37 °C in a multicomponent buffer (100 mM acetate, 100 mM MES, 100 mM HEPES, 100 mM CHES, 100 mM CAPS).

**Table 3:** Observed first-order rate constants (k_obs_) for the single turnover conversion of Ac-LKRR-*p*Na substrate by wild type and mutant forms of both the glycine-linked and unlinked NS2B-NS3pro complexes at 37 °C, pH 8.5, with a 20% excess of enzyme.

	Glycine-linked	Unlinked
k_obs (_*s*^*−1*^)		
WT	0.82 ± 0.31^a^	0.88 ± 0.20
L74A	0.69 ± 0.50	1.04 ± 0.70
I76A	0.62 ± 0.31	0.98 ± 0.51
E80A	0.99 ± 0.70	0.70 ± 0.11
S83A	0.78 ± 0.62	0.63 ± 0.22
I86A	0.61 ± 0.30	0.52 ± 0.13

^a^For the WT, glycine-linked protease, data was also collected at nearly three- and five-fold excess of enzyme. Corresponding first-order rate constants obtained were 0.79 ± 0.4 s^−1^ and 0.81 ± 0.3 s^−1^, respectively, demonstrating that single turnover conditions are attained with a 20% excess of enzyme.

**Table 4:** Comparison of steady state (k_cat_, K_m_, k_cat_/K_m_) and pre-steady state (k_obs_, k_1_, k_2_) kinetic parameters for wild-type and Ser83Trp mutant forms of the glycine-linked and unlinked NS2B_H_-NS3pro complexes, using the substrate Ac-LKRR-*p*Na at 37 °C, pH 8.5.

	K_m_ (*μM)*	k_cat_ (*s*^*−1*^)	k_cat_/K_m_ (_M_^−1^s^−1^)	k_obs_ (*s*^*−1*^)	k_1_ *(s*^*−1*^)	k_2_ *(s*^*−1*^)
Glycine-linked WT	282 ± 22	0.34 ± 0.01	1191 ± 104	0.82 ± 0.31	12 ± 1	—
Glycine-linked S83W	624 ± 69	1.17 ± 0.09	1880 ± 252	0.78 ± 0.43	15 ± 3	350 ± 23
Unlinked WT	197 ± 21	0.46 ± 0.02	2345 ± 276	0.88 ± 0.23	112 ± 11	—
Unlinked S83W	215 ± 17	0.47 ± 0.01	2180 ± 190	0.58 ± 0.20	52 ± 2	210 ± 32
