# Supplementary material for: Product release is rate-limiting for catalytic processing by the Dengue virus protease
Source: Sci Rep. 2016 Nov 29;6:37539. doi: 10.1038/srep37539 (PMC5126634; doi:10.1038/srep37539)
Supplement: Supplementary Information [file srep37539-s1.pdf]

SUPPLEMENTARY DATA

Product release is rate-limiting for catalytic processing by Dengue virus protease

Shannon, A.E., Pedroso, M.M., Chappell, K.J., Watterson, D., Liebscher, S., Kok, W.M., Fairlie, D.P., Schenk, G., Young, P.R.

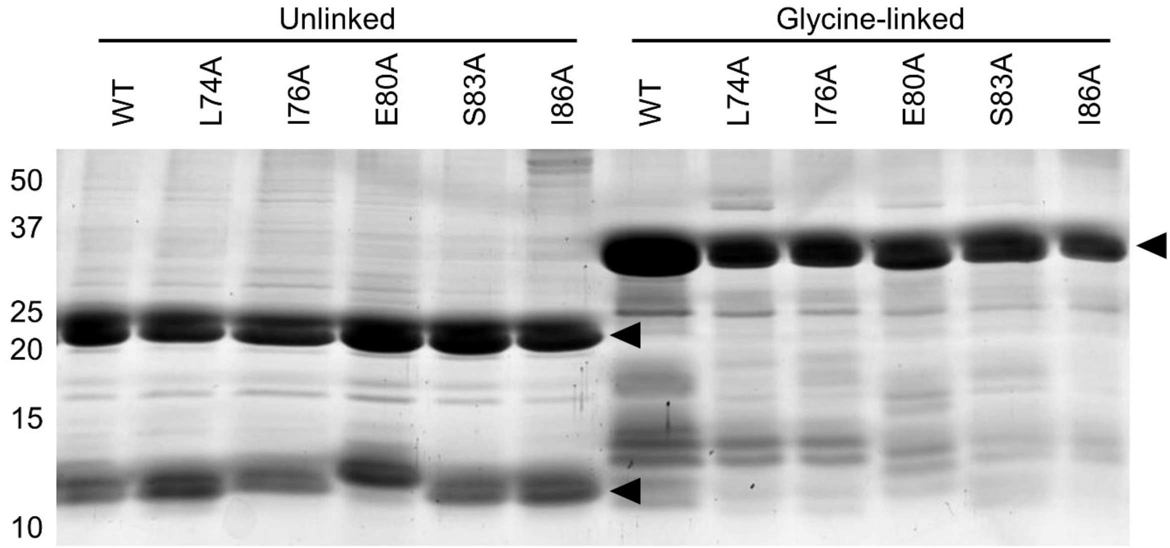

Figure S1. Expression profiles of mutant NS2B$_H$-NS3pro. Coomassie-stained SDS-PAGE of wild-type and mutant NS2B$_H$-NS3pro complexes expressed in *E.coli* using two expression systems. MW in kDa is indicated on the left. For the unlinked complexes, the top arrow is NS3pro and bottom arrow is 6His-NS2B$_H$. For the glycine-linked proteases, the arrow is 6His-NS2B$_H$-G$_4$SG$_4$-NS3pro.

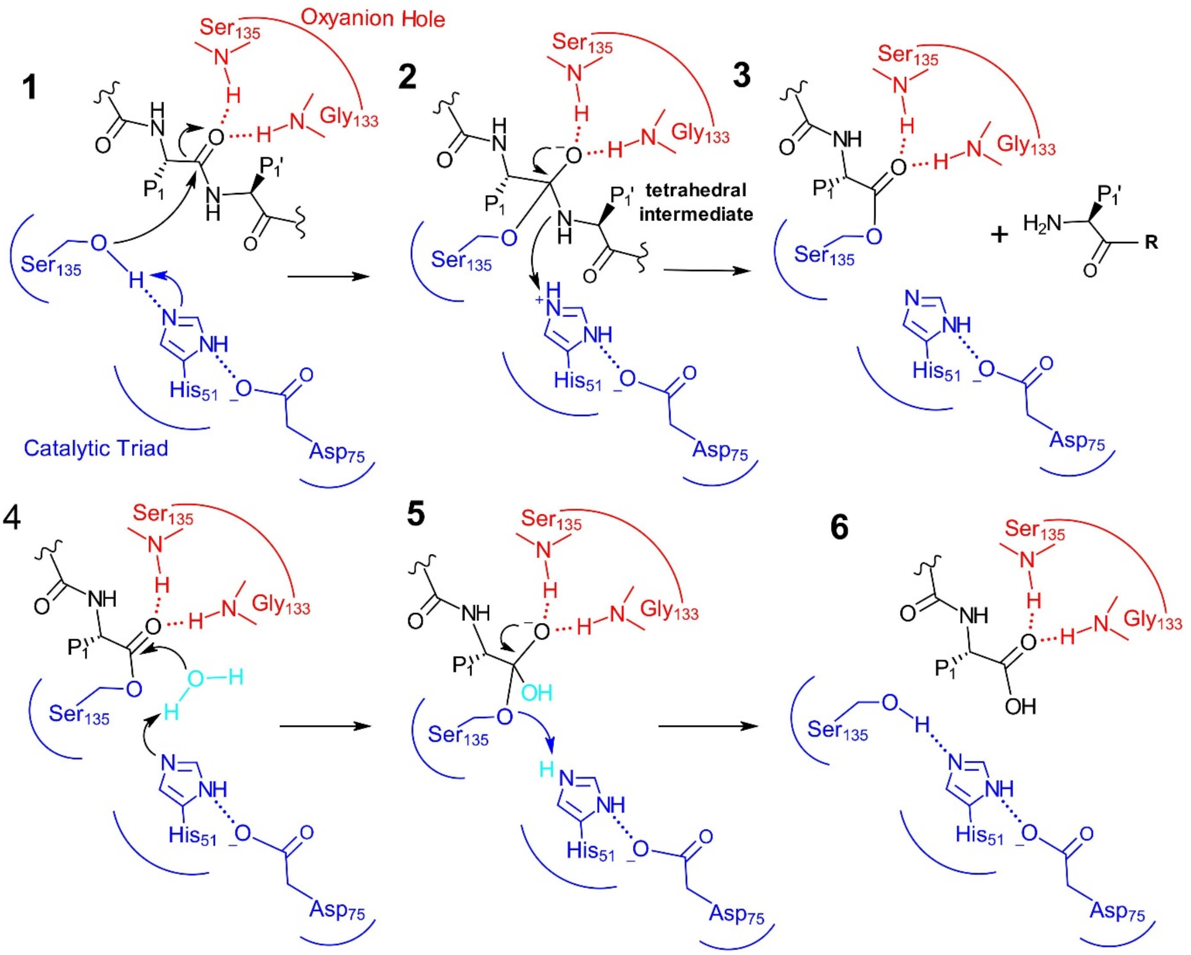

Figure S2. Mechanism of Substrate hydrolysis by DENV NS2B-NS3 protease. 1) The oxygen of active-site serine-135 undergoes nucleophilic attack on carbonyl carbon of the substrate 2) A short-lived tetrahedral intermediate oxyanion is formed, stabilised by oxyanion interaction with two hydrogen bonding backbone amide NH protons of Ser135 and Gly133 residues that border the oxyanion hole 3) The tetrahedral intermediate collapses, forming an acyl-enzyme intermediate and releases an amine product protonated by His57. 4) A water molecule is activated by His51 for nucleophilic attack on the acyl intermediate. 5) A second tetrahedral intermediate is temporarily formed. 6) The Ser135 oxygen is protonated by the imidazole of His51 with collapse of the tetrahedral intermediate, release of the carboxylate product and restoration of the catalytic site in its substrate-binding form.

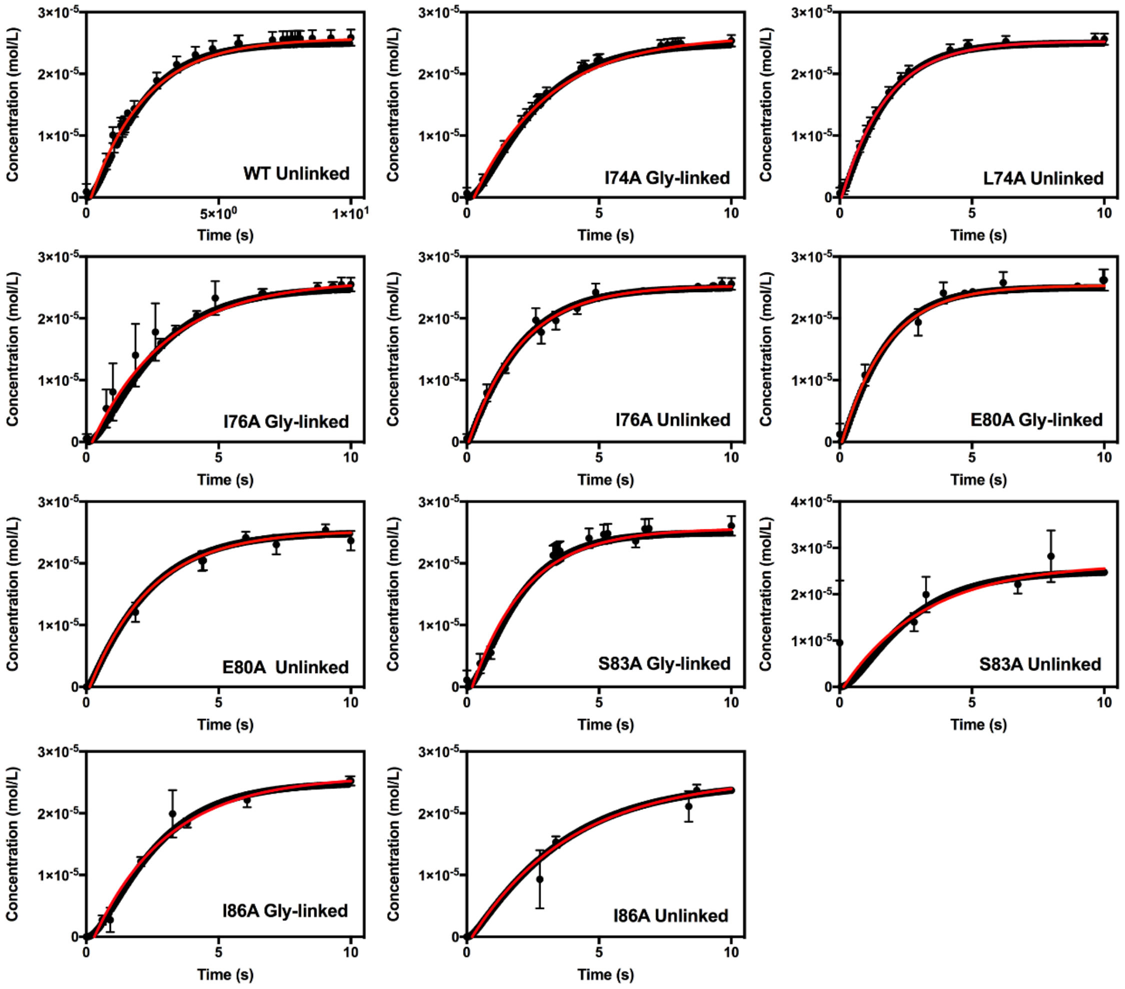

Figure S3. Single turnover enzyme kinetics, time course for product formation. Hydrolysis of the *para*-nitroanalide substrate Ac-LKRR-*p*Na (25 µM) by the wild-type form of the unlinked DENV2 protease complex, and all $NS2B_H$ mutants in both the glycine-linked and unlinked systems. Activity measured in 50 mM Tris.HCl, pH 8.5 at 37 °C. The data were fitted to a first-order exponential (providing an estimate of k_{obs}) using Reactlab software (eq. 3).
